# Accuracy and precision of the RABBIT technique

**DOI:** 10.1098/rsta.2017.0475

**Published:** 2019-04-01

**Authors:** M. Isinger, D. Busto, S. Mikaelsson, S. Zhong, C. Guo, P. Salières, C. L. Arnold, A. L'Huillier, M. Gisselbrecht

**Affiliations:** 1 Department of Physics, Lund University, PO Box 118, 22 100 Lund, Sweden; 2 LIDYL, CEA, CNRS, Université Paris-Saclay, CEA Saclay, 91191 Gif-sur-Yvette, France

**Keywords:** attosecond physics, high-order harmonic generation, RABBIT, photoionization timedelays

## Abstract

One of the most ubiquitous techniques within attosecond science is the so-called reconstruction of attosecond beating by interference of two-photon transitions (RABBIT). Originally proposed for the characterization of attosecond pulses, it has been successfully applied to the accurate determination of time delays in photoemission. Here, we examine in detail, using numerical simulations, the effect of the spatial and temporal properties of the light fields and of the experimental procedure on the accuracy of the method. This allows us to identify the necessary conditions to achieve the best temporal precision in RABBIT measurements.

This article is part of the theme issue ‘Measurement of ultrafast electronic and structural dynamics with X-rays’.

## 1. Introduction

The study of ultrafast dynamics of small quantum systems is currently undergoing rapid progress due to the emergence of intense and ultra-short pulsed light sources in the extreme ultraviolet (XUV) and X-ray regimes. One driving force behind this progress is the field of attosecond science, thanks to light sources based on high-order harmonic generation (HHG) in gases, allowing the investigation of electron dynamics at the fastest available time scale [1]. A world-spanning effort has led to the generation of pulses with duration below 100 as [2–5], photon energies up to several kiloelectronvolts [6,7] and pulse energies up to a few microjoules [8,9], enabling the exploration of real-time dynamics of fundamental processes induced by light–matter interaction, such as photoionization [10]. To push further the frontier of attosecond science and reach the ultimate temporal resolution, the metrology of the available experimental techniques needs to be addressed.

Two major techniques, commonly referred to as streaking [11–13] and RABBIT (reconstruction of attosecond beating by interference of two-photon transitions) [14–17], are currently used to get an insight into the attosecond dynamics of photoemission. Both techniques are based on cross-correlation measurements between the attosecond XUV light field and a phase-locked infrared (IR) laser field. The measurements consist in producing electron wave-packets, whose temporal characteristics essentially mimic those of the ionizing XUV light field. The electron wave-packets are thereafter probed, in the vicinity of the ionic core, by an IR field of intensity varying from 10^11^ to 10^13^ W cm^−2^. Theoretical works have provided a deep understanding of the temporal properties of these electron wave-packets, allowing one to disentangle the contributions of the light fields and of the photoionization dynamics [18,19]. However, from an experimental perspective, the temporal information that can be extracted from the electron wave-packets is often limited by the precise knowledge (or control) of the light properties, including temporal and spatial overlap, and the performance of the numerical algorithms used to analyse the data.

Streaking uses a single attosecond pulse and an intense IR field to streak the photoelectrons at different times. Various reconstruction methods to characterize these ultra-broadband attosecond pulses have been proposed and implemented [5,20–23] and rigorous studies have discussed their robustness [5,24–27]. RABBIT relies on an attosecond pulse train and a weak probe field to induce interference between multiple quantum paths in the spectral domain. The RABBIT technique has been compared to streaking in an extensive study [28], as well as to two-photon volume autocorrelation [29]. To our knowledge, as of yet, there is no systematic investigation of the problems inherent to RABBIT measurements that could limit the accuracy of the technique, and of the ultimate temporal precision that can be reached.

In this work, we present a comprehensive analysis of the RABBIT technique. Its principle is described in §2 and we investigate the influence of the properties of the light fields in §3. We finally discuss the statistical effects inherent to any measurement in order to define optimal experimental parameters in §4.

## 2. Theory and experiment

### (a) Principle

**Figure 1.**
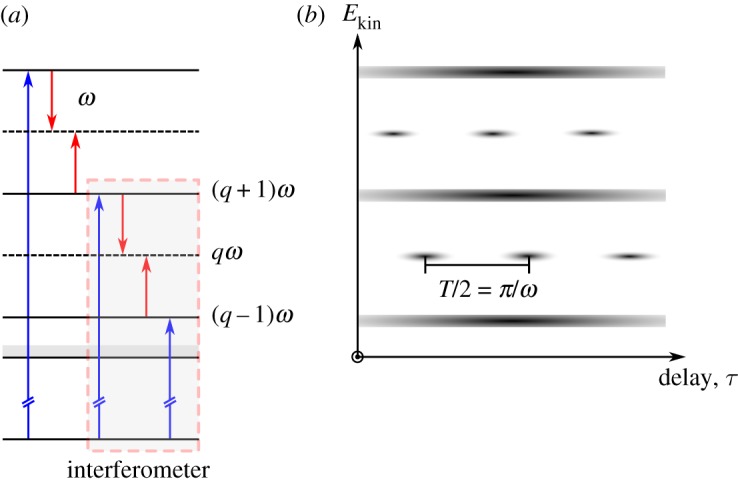
Principle of RABBIT. Each sideband *q* makes up a quantum interferometer, one arm being the absorption of harmonic *q* − 1 followed by absorption of an IR photon, and the other the absorption of harmonic *q* + 1 followed by emission of an IR photon, leading to the same final energy. The intensity of the sideband oscillates as a function of the delay between the pulses, with an oscillation period of *T*/2, where *T* is the period of the fundamental, and the phase of this oscillation reveals information about the target system. (Online version in colour.)

The principle of the RABBIT technique is presented in figure 1. An XUV attosecond pulse train (APT), corresponding in the frequency domain to a comb of odd-order harmonics, is overlapped spatially and temporally with an IR pulse which is a weak replica of the field used to generate the APT, and phase-locked to it. The interaction of atoms or molecules with both light fields leads to the creation of electron wave-packets, composed of peaks due to ionization by the XUV APT and sidebands, which arise from two-photon transitions (XUV ± IR). The sideband signals oscillate as the delay between the IR and XUV pulses is varied. This is due to an interference between two quantum paths, the first one involving absorption of one harmonic and an IR photon, and the second involving absorption of the next harmonic and emission of an IR photon. We use second-order perturbation theory for the light–matter interaction, expressed with the dipole approximation, make the rotating-wave approximation, considering only the processes where the XUV fields are absorbed first, and assume that the fundamental and harmonic fields are monochromatic (the latter assumption will be removed in §3b). For a single atom/molecule, the *q*th sideband signal can be described by the equation [14]

2.1 $$\begin{array}{rl} S_{q} & =|A_{\mathrm{abs}}+A_{\mathrm{emi}}{|}^{2}\\ & =|A_{\mathrm{abs}}{|}^{2}+|A_{\mathrm{emi}}{|}^{2}+2|A_{\mathrm{abs}}||A_{\mathrm{emi}}|\cos\left[\arg(A_{\mathrm{abs}})-\arg(A_{\mathrm{emi}})\right], \end{array}$$

where $A_{\mathrm{abs}}=E_{1}E_{q-1}M_{\mathrm{abs}}$ and $A_{\mathrm{emi}}=E_{1}^{*}E_{q+1}M_{\mathrm{emi}}$. In these expressions, $E_{1}$ and $E_{q\pm 1}$ denote the amplitudes of the fundamental and (*q* ± 1)th harmonic fields, while *M*_abs_ (*M*_emi_) are two-photon ionization matrix elements involving absorption (emission) of the fundamental field in the continuum. It is often assumed that the phase of the fundamental field is constant, and simply related to that of the APT by a variable delay *τ*, so that $\arg(E_{1})-\arg(E_{1}^{*})=2\omega \tau$, *ω* denoting the fundamental frequency (*T*/2 = *π*/*ω* ≈ 1.3 fs at 800 nm). We further denote $\arg(E_{q+1})-\arg(E_{q-1})=2\omega \tau_{\mathrm{XUV}}$ and $\arg(M_{\mathrm{emi}})-\arg(M_{\mathrm{abs}})=2\omega \tau_{A}$, so that equation (2.1) becomes

2.2 $$S_{q}=|A_{\mathrm{abs}}{|}^{2}+|A_{\mathrm{emi}}{|}^{2}+2|A_{\mathrm{abs}}||A_{\mathrm{emi}}|\cos[2\omega (\tau -\tau_{R})],$$

where *τ*_R_ = *τ*_xuv_ + *τ*_A_. The first term is the group delay of the attosecond pulses in the train, which is here assumed to be perfectly periodic, and the second term comes from the phase difference between the two-photon matrix elements [15,18]. An accurate determination of *τ*_R_ requires good control over the delay *τ*. In the experiments, the delay *τ*_R_ is obtained through an analysis of RABBIT spectrograms (figure 1*b*), which implies a volume integration over the interaction region, according to

2.3 $$S_{q}=\int \left(|A_{\mathrm{abs}}{|}^{2}+|A_{\mathrm{emi}}{|}^{2}+2|A_{\mathrm{abs}}||A_{\mathrm{emi}}|\cos[2\omega (\tau -\tau_{R})]\right)\;d^{3}r.$$

The dependence of the amplitudes $A_{\mathrm{abs}}$ and $A_{\mathrm{emi}}$ on space do not affect the phase of the oscillation and therefore not the precision of the RABBIT measurement. However, when *τ*_R_ depends on space over the region of overlap, the sideband oscillations become blurred, leading to an inaccuracy in the phase retrieval. One aim of this article is to study the limitations to the measurement induced by spatial variations of the XUV field.

To eliminate the influence of the excitation pulse, two measurements can be performed simultaneously, for example, on different ionization processes [10,30–34] or in different target species [35–38]. This enables the determination of a relative photoionization time delay Δ*τ*_A_. The absolute photoionization delay *τ*_A_ can be deduced if we assume that one of the delays can be sufficiently accurately calculated to serve as an absolute reference [34,39].

Originally, the RABBIT technique has consisted in analysing the energy-integrated oscillations of the sideband signal. State-of-the-art photoelectron spectrometers now provide high spectral resolution which allows for an *energy-resolved* analysis of the sideband oscillations. This technique, dubbed rainbow RABBIT [40], can be used to retrieve the amplitude and phase variation across sidebands, which is useful to disentangle different ionization processes contributing to the same sideband [10,41], or to characterize complex photoelectron wave-packets [40,42,43].

### (b) Experimental methods

Various experimental set-ups have been designed for the RABBIT technique [15,17,44,45]. The common approach is to use a Mach–Zehnder-type interferometer [46,47] to control the delay between the pump and the probe beams. A requirement for passive stabilization is that the parity of the number of mirrors in each arm be the same, which avoids lateral fluctuations. Often, active stabilization, with a delay stage controlled by a feedback loop that monitors the delay and accounts for drifts [48], is preferred. This ensures both short-term and long-term stability of the delay between the two arms [47,49]. However, the jitter between the pump and probe arms cannot be fully corrected and typical values of this delay jitter ranges from 200 as r.m.s. (passive stabilization) down to 50 as r.m.s. (active stabilization) [48]. With the recent implementation of a beam pointing stabilization technique to account for any spatial drifts that occur on a rate of 100 Hz or slower, we can currently reach delay jitters of the order of 25 as [49]. Efforts are being made to improve these values even further.

Once the experiment is carried out, the spectrogram needs to be post-processed in order to extract the phase of individual sidebands. One approach is to perform Fourier analysis to efficiently extract the oscillation frequencies present in the spectrogram and then analyse the raw traces using a robust nonlinear Levenberg–Marquardt algorithm [50,51]. The algorithm returns the best-fit phase as well as an estimate of the goodness of fit, essentially given by the statistical properties of the signal.

### (c) Simulations

**Figure 2.**
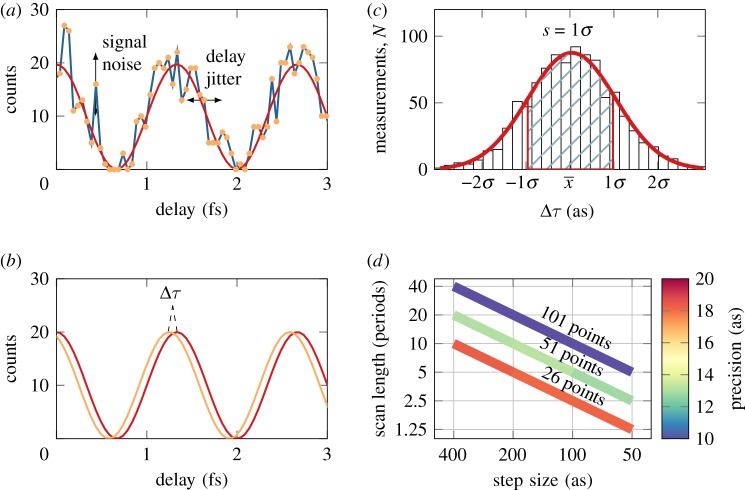
Illustration of the model used to simulate RABBIT traces. (*a*) A RABBIT oscillation, on which temporal jitter and signal fluctuations are added; (*b*) determination of the phase difference between the best fit and the original function; (*c*) after repetition of this procedure *N* times, the precision can be determined as the standard deviation of the distribution function; (*d*) precision (in colour) as a function of scan length and step size for different number of sampling points, with a temporal jitter, *σ*_*t*_, of 25 as. No signal and background noise was taken into account.

Finally, we present how we model RABBIT measurements, using Monte Carlo simulations. A sample is obtained by discretizing equation (2.2) for a certain number of periods and a given step size, as seen in figure 2*a*. Each sample is then perturbed by an insertion of noise in both dimensions, to emulate interferometer instability and statistical noise. The temporal jitter is drawn from a normal distribution with a variance *σ*^2^_*t*_ of a pre-set value. The jitter of the electron signal is drawn from a Poisson distribution with a variance *σ*^2^_*s*_, equal to the acquired counts at each step. The noisy signal is then used as input for the nonlinear least-squares fit (figure 2*b*), and the phase difference between the best fit and the original unperturbed cosine is evaluated, giving the error of the fitted phase. This process is repeated *N* times to acquire enough statistics to reveal a normal distribution of values around the mean value $\bar{x}=\sum_{i=1}^{N}x_{i}/N$, as shown in figure 2*c*. The normal distribution is fitted and the precision, defined as the sample standard deviation *s*, can be obtained as

2.4 $$s=\sqrt{\frac{1}{N-1}\sum_{i=1}^{N}{\left(x_{i}-\bar{x}\right)}^{2}}.$$

Figure 2*d* shows how the precision varies as a function of scan length and step size. The step size is decreasing as the scan length is decreasing, so as to keep the number of sampling points constant. It is of great importance to realize that it is the total number of sampling points that determines the temporal precision with this phase retrieval algorithm, as long as the oscillation frequency is resolved.

Note that the precision should not to be confused with the accuracy (often called standard error of mean or s.e.m.), which would be a value of how close the estimated mean from a set of *N* RABBIT measurements is to the true mean, and scales as $s/\sqrt{N}$. To approach the true mean, we can either increase the precision, i.e. minimize *s*, of each measurement or acquire a larger set, *N*, of data, as long as there are no systematic errors. Ultimately, a good RABBIT measurement requires both high precision and high accuracy.

## 3. Influence of the spatial and temporal properties of the light fields

In this section, we investigate the influence of the properties of the harmonic radiation on the accuracy of the extracted time delay from the RABBIT technique, defined as the difference between the measured value and the real one.

### (a) Spatial variations of the RABBIT phase

The (*q* ± 1)th harmonic field has a phase equal to (*q* ± 1)*ϕ*_fund_ + *Φ*_*q* ± 1_, where *ϕ*_fund_ is the phase of the fundamental field used for the generation of high-order harmonics and *Φ*_*q* ± 1_ is the dipole phase, accumulated by the electron on its trajectory. For a Gaussian beam, the spatial phase is *ϕ*(*r*, *z*) = *kz* + *kr*^2^/*R*(*z*) − *ζ*(*z*), where *R*(*z*) = *z*(1 + *z*^2^_0_/*z*^2^) is the radius of curvature and $\zeta (z)=\arctan(z/z_{0})$ the Gouy phase (*z*_0_ is the Rayleigh length). The phase entering the RABBIT equation is

3.1 $$2\arg(E_{1})-[\arg(E_{q+1})-\arg(E_{q-1})]=(2\omega \tau +2\phi_{\mathrm{probe}})-(2\phi_{\mathrm{fund}}+\Delta \Phi_{q}),$$

where *ϕ*_probe_ is the phase of the probe field in the interaction (detection) region and Δ*Φ*_*q*_ = *Φ*_*q*+1_ − *Φ*_*q*−1_.

Let us assume that the probe field and fundamental field generating the harmonics have the same phase. This requires that the same focusing optics is used in both probe and pump arms. Imperfect focusing of the attosecond light may induce a spreading of the radiation both in space and in time due to optical aberrations [52–54], leading to distortions of the corresponding RABBIT traces. In the following, we do not consider such effects. To include the influence of the dipole phase, we use an analytic expression obtained by linearizing the variation of the emitted frequency with return time for each of the trajectories contributing to the harmonic emission [55,56]. For the short trajectory used in RABBIT measurements,

3.2 $$\Phi_{q\pm 1}=\frac{\gamma }{I}{\left(q\pm 1-q_{p}\right)}^{2}\omega^{2},$$

where *γ* is a gas-independent coefficient equal to 1.03 × 10^−18^ s^2^ W cm^−2^ at 800 nm, *q*_*p*_ = *I*_*p*_/*ω*, where *I*_*p*_ is the ionization potential of the generating gas, and *I* is the laser intensity. The difference of phase between the two quantum paths entering equation (2.2) due to the harmonic fields is therefore

3.3 $$\Delta \Phi_{q}=\frac{4\gamma }{I}(q-q_{p})\omega^{2}.$$

This expression gives a simple estimate of the group delay of the attosecond pulses, also called ‘attochirp’,

3.4 $$\tau_{\mathrm{XUV}}=\frac{\Delta \Phi_{q}}{2\omega }=\frac{2\gamma }{I}(q-q_{p})\omega ,$$

which varies linearly with the harmonic order, except close to the cutoff frequency [17,57].

**Figure 3.**
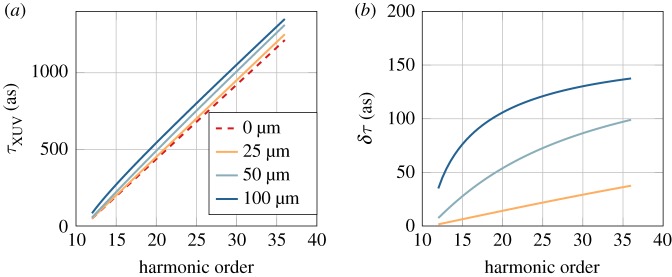
Effect of the spatial variation of the light fields. (*a*) ‘Measured’ group delay obtained by a RABBIT measurement integrated over the focal plane for different XUV beam waists as a function of the sideband order. (*b*) Difference *δτ* between the ‘measured’ group delay and the group delay at *r* = 0 for different XUV beam waists as a function of sideband order.

This expression also shows that *τ*_xuv_ depends on the radial coordinate, *r*, through the variation of the laser intensity *I*,

3.5 $$\tau_{\mathrm{XUV}}(r)=\frac{2\gamma (q-q_{p})\omega }{I_{0}}\exp\left(\frac{2r^{2}}{w^{2}}\right),$$

where we assume that the laser is Gaussian, with *I*_0_ the laser peak intensity and *w* the radius at 1/e^2^. We can now estimate the influence of *r*-dependent attochirp on the group delay retrieval in a RABBIT measurement. Assuming no further distortion of the phase of the XUV field from the generation to the detection, we can insert this phase variation in equation (2.3) and calculate its influence on the RABBIT traces, using a radius *w* = 100 μm and an intensity *I*_0_ = 10^14^ W cm^−2^ (this intensity will be used for all the simulations throughout the article). Figure 3*a* shows the group delay obtained from RABBIT measurements, integrated over the focal plane for different XUV radii. The observed effect is relatively small compared to the attochirp. As expected, it increases with the harmonic order. Figure 3*b* shows the difference between the ‘measured’ group delays and the group delay at *r* = 0.

This effect should not, however, affect RABBIT measurements, consisting of two experiments performed simultaneously. This explains why the spatial dependence of the phase of the fields in equation (2.1) has not been discussed in RABBIT measurements, though the importance of wavefront matching of the pump and probe beams has been pointed out [44]. If the focusing geometries for the probe and harmonic beams are, however, different, not only errors due to wavefront mismatch may appear, but also the terms including the Gouy phase do not necessarily cancel out and need also to be considered in the error budget. In addition, possible delays between the harmonic fields and the probe field on the way to the application chamber, due to reflections on focusing mirrors for example, should also be taken into account [39,58].

### (b) Influence of the temporal properties of the light fields

The dipole phase has two effects on the temporal properties of the XUV radiation. It leads to a positive group delay of the attosecond pulses (see equation (3.4)). It also leads to a femtosecond chirp of the individual harmonics (in the frequency domain, to group delay dispersion) [59].

For a Gaussian pulse with intensity profile $I(t)=I_{0}\exp[-at^{2}/\tau^{2}]$, where *a* = 4ln(2) and *τ* is the pulse duration (full width at half-maximum), equation (3.2) can be approximated by

3.6 $$\Phi_{q\pm 1}\approx \frac{\gamma {\left(q\pm 1-q_{p}\right)}^{2}\omega^{2}}{I_{0}}\left(1+\frac{at^{2}}{\tau^{2}}\right).$$

The chirp coefficient, so-called chirp rate, is

3.7 $$b_{q\pm 1}=-\frac{\partial^{2}\Phi_{q\pm 1}}{\partial t^{2}}\approx -\frac{2a\gamma {\left(q\pm 1-q_{p}\right)}^{2}\omega^{2}}{\tau^{2}I_{0}}.$$

The corresponding group delay dispersion (GDD), *ϕ*′′_*q* ± 1_, is related to the chirp rate by

**Figure 4.**
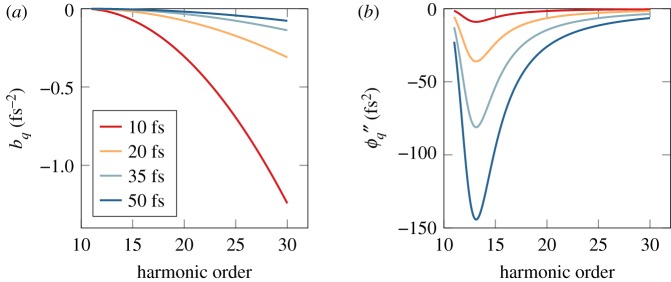
Intrinsic femtosecond chirp of the XUV radiation. Chirp rate (*a*) and group delay dispersion (*b*) of individual harmonic for different pulse durations of the fundamental laser.

3.8 $$\phi_{q\pm 1}^{\prime \prime }=\frac{b_{q\pm 1}\tau_{q\pm 1}^{4}/2}{a^{2}+b_{q\pm 1}^{2}\tau_{q\pm 1}^{4}},$$

where *τ*_*q* ± 1_∼*τ*/2 is the full width at half-maximum pulse duration of the (*q* ± 1)th harmonic [60], also assumed to be Gaussian. The chirp coefficient and the corresponding group delay dispersion for different harmonics order are plotted in figure 4*a*,*b* for different fundamental laser pulse durations.

**Figure 5.**
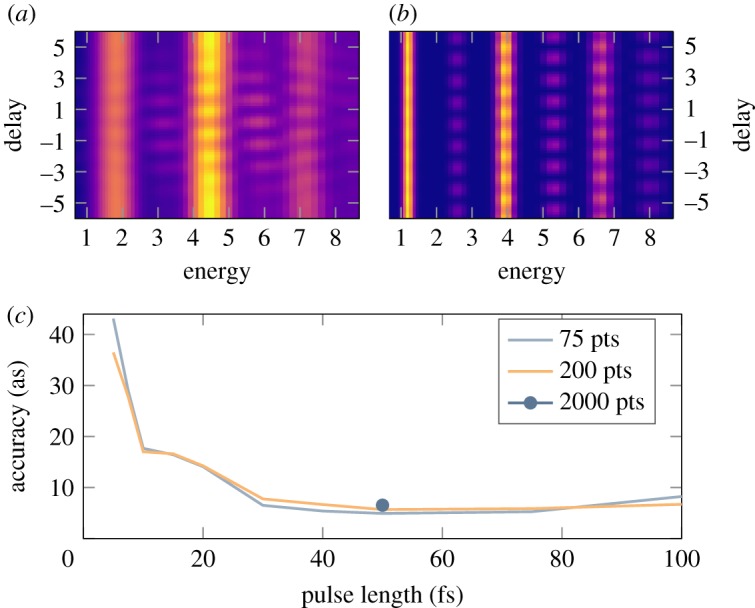
Effect of the chirp rate as a function of the fundamental pulse duration in RABBIT measurement. (*a*,*b*) Simulated scans in helium are exhibited for pulses of 5 fs and 30 fs, respectively. (*c*) The error in the phase retrieval for sideband 19 (at 5–6 eV kinetic energy). To ensure the convergence of the results, simulations were performed by varying the number of sampling points.

As can be seen in figure 4*a*, the chirp coefficient decreases monotonically as the harmonic order increases. This implies that the difference in time between attosecond pulses is not exactly equal to *T*/2. In a traditional RABBIT experiment, this effect introduces correction terms to equation (2.2) and in particular a dependence on the delay due to the difference in chirp rates between consecutive harmonics [61]. While these correction terms can be considered negligible for long pulses, they become important for short pulses. To evaluate the error made in the phase retrieval using equation (2.2) as a function of the fundamental pulse duration, we performed strong-field approximation (SFA) simulations of RABBIT scans in helium. The results of the simulations are presented in figure 5. Three regions can be identified: below 10 fs, between 10 and 30 fs and above 30 fs. At the shortest pulses, large errors are observed due to the lack of periodicity between the minima. Note that an algorithm that also fitted the autocorrelation envelope of the sideband signal returned the same results, assuring us that the effect at short pulse lengths is not related to the fit model. The error decreases until it reaches a plateau for pulse duration above 30 fs. The height of the plateau of about 5 as reflects the inherent error made by not including the chirp rate in equation (2.2). The latter is essentially removed when performing two measurements simultaneously. Finally, it is worth mentioning that all correction terms to equation (2.2) depend on the chirp rate and are therefore sensitive to intensity fluctuations of the fundamental laser. We estimated with SFA simulations that the temporal accuracy is degraded by approximately 60% for intensity fluctuations of the fundamental laser of typically 5% and up to a factor of two for intensity fluctuations of 25%. These effects related to intensity fluctuations cannot be completely removed by performing two measurements at the same time.

**Figure 6.**
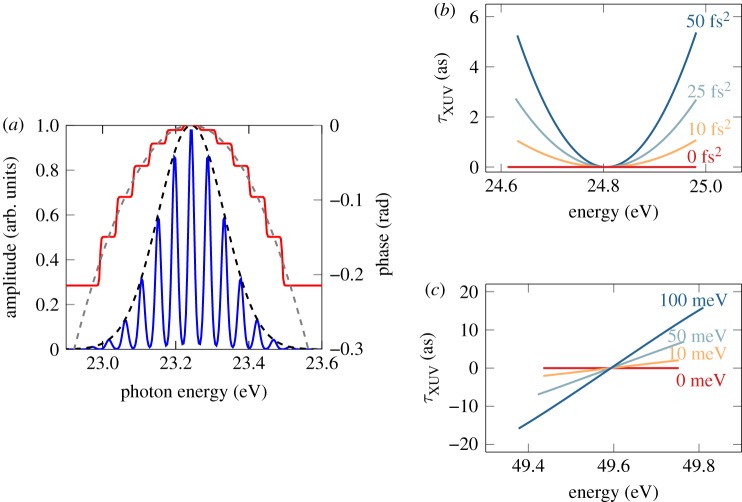
Intra-sideband delay variation. (*a*) Numerical decomposition of harmonic 15. The dashed black curve is the amplitude of the pulse and the dashed grey curve is the phase. The pulse is decomposed into 13 different subpulses, each with a different constant phase. The blue curve shows the resulting amplitude and the red curve the resulting phase. (*b*) Delay variation in sideband 16 for different values of Δ*ϕ*_*q*_′′. There is no blueshift. (*c*) Delay variation in sideband 32 for different blueshifts. The femtochirp of both harmonics is the same (−50 fs^2^). Note that in this case, the different phases do not span the same energy, as sidebands are broadened by the blueshift. In (*b*, *c*), the harmonic bandwidth is set to 150 meV and the probe bandwidth to 70 nm. The energy range is limited by a threshold of 30% of the maximum sideband intensity, below which we consider that the phase cannot be reliably extracted [43].

We examine now the influence of the femtosecond chirp on the phase retrieval in the rainbow RABBIT method. Since the sidebands result from the interference of multiple combinations of IR and XUV frequencies, the spectral characteristics of the light fields become important. A rigorous theoretical treatment of rainbow RABBIT requires a model that goes beyond the approximation presented so far. Here, we adapt the analytic two-photon finite-pulse model developed by Jiménez-Galán *et al*. [62] to study the influence of chirped XUV pulses on the retrieved phase from a rainbow RABBIT measurement. Since the original finite-pulse model assumes Fourier limited pulses, we numerically decompose the XUV pulse as a sum of 13 subpulses each with a different constant phase as shown in figure 6*a*, where the dashed curves correspond to the initial amplitude and phase while the solid curves represent the result of the numerical decomposition. This decomposition allows us to include the phase variation over the harmonic bandwidth, in particular due to the group delay dispersion, which is strongly dependent on the harmonic order. The two-photon transition amplitudes are then computed independently for each subpulse and are coherently added. For the calculation, we consider two consecutive harmonics with a bandwidth of 150 meV and a Fourier-limited IR probe pulse with a bandwidth of 70 nm. In the absence of resonant states, the measured delay variations can be mainly attributed to *τ*_xuv_.

Two different cases are presented using the results of figure 4*b*. The first one, in figure 6*b*, is for low-order harmonics (*q* < *q*_*p*_ + 10), where the variation of the GDD as a function of harmonic order, Δ*ϕ*′′_*q*_ = *ϕ*′′_*q*+1_ − *ϕ*′′_*q*−1_, can be important. The second one, in figure 6*c*, is for high harmonic orders (*q* > *q*_*p*_ + 10), where Δ*ϕ*′′_*q*_ varies slowly as a function of the harmonic order, and can be neglected. In the first case, the measured delay *τ*_xuv_ can exhibit a quadratic behaviour as a function of electron kinetic energy *E* over the sideband bandwidth due to the difference in GDD between the consecutive harmonics. This effect, in the case of standard energy-integrated RABBIT measurements, introduces a systematic error which can affect the accuracy of the technique if it is not taken into account when measuring the atomic delays *τ*_A_. However, if both harmonics have a similar GDD, the spectral phases of the two harmonics compensate each other, giving rise to a constant *τ*_xuv_ over the sideband bandwidth, which is a common valid assumption for high harmonic orders.

However, even in the case where both harmonics have the same GDD, the delay may vary over the sideband bandwidth if the contributions from the higher and lower harmonics to the sideband do not perfectly overlap spectrally. Indeed, the IR pulses used to generate harmonics are intense enough so that the front of the pulse can partially ionize the medium through which the rest of the pulse propagates, causing a blueshift of the IR frequency used to generate the harmonics [63]. In most experimental settings, the probe pulse does not propagate through the plasma. The contributions from the lower and higher harmonics will therefore be shifted with respect to each other. It has been shown in [43] that this effect leads to a linear phase variation which is proportional to the blueshift. In figure 6*c*, we show the influence of the laser blueshift on the retrieved delay. The GDD of the two neighbouring harmonics is the same (−50 fs^2^) but the fundamental frequency of the laser pulse used to generate the harmonics is blueshifted while keeping the central frequency of the probe pulse at ℏ*ω* = 1.55 eV. A blueshift of the central frequency of the generating IR pulse leads to a non-optimal overlap of the contributions of the consecutive harmonics which results in a quasi-linear phase variation over the sideband, in qualitative agreement with the predictions of [43]. However, the delay extracted from the energy-integrated sideband signal will not be affected by this linear phase variation. In general, both GDD variation and blueshift of the fundamental contribute to the total variation of *τ*_xuv_ over the sideband. The magnitude of these effects increases with the duration of both IR and harmonic fields. Similarly, any chirp of the probe pulse may perturb (rainbow) RABBIT measurements and would have to be taken into account. It is therefore important to monitor the generation conditions to be able to take into account these effects when retrieving the atomic delays.

## 4. Optimization of RABBIT measurements

Finally, we discuss the influence of fluctuations and statistics on RABBIT measurements in order to optimize the experimental settings (e.g. step size for sampling equation (2.1) versus scan length). Note that a different type of data analysis, named mixed-FROG (frequency-resolved optical gating), has been proposed in this context in order to access the partial coherence of an ensemble of atoms [64]. We here stay in the frame of the ‘standard’ RABBIT analysis.

### (a) Influence of the temporal jitter

In a RABBIT measurement, the temporal jitter arises due to a combination of air flow affecting the optical path in each arm, thermal expansion of the mirrors in the arms of the interferometer, and beam pointing instability, which cannot be accounted for by the active stabilization loop. These sources of jitter occur on a time scale comparable to the acquisition time (seconds to minutes) and we evaluate how they affect the precision of the retrieved phase, by running a set of Monte Carlo simulations as described in §2d. The parameter space investigated in our simulations consists of the *sampling step size*, the *scan length* and interferometric *stability*, which was set to different values during the simulations. If we neglect any effects related to the femtochirp, we need to sample equation (2.2) with a finite sampling rate and a finite number of periods. An error is immediately introduced due to the finite sampling; we therefore fixed two parameters and analysed the standard deviation of the error (i.e. the precision) as a function of the third parameter.

**Figure 7.**
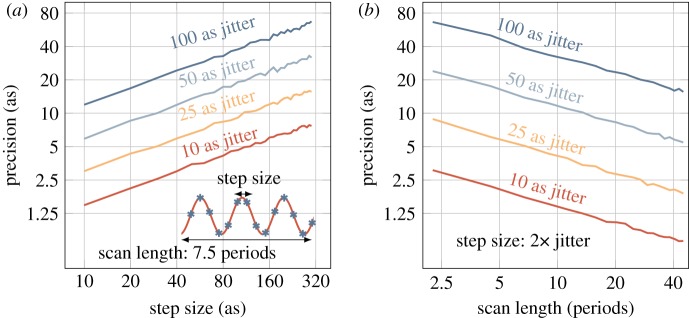
RABBIT precision extracted from different sets of 1000 Monte Carlo simulations. (*a*) The temporal jitter was set to the four values indicated and the precision was calculated while the step size was decreased. (*b*) The temporal jitter was set to the four values indicated, and the step size was set to two times the magnitude of the jitter. The precision was calculated while the number of periods was increased. One period in this case was set to 1.33 fs (i.e. 800 nm wavelength).

The step size was first varied between 300 as (i.e. just about four points per cycle) and 10 as (over 130 points per cycle) for four different jitter amplitudes, ranging from 100 as (the r.m.s. of a good passively stabilized interferometer) to 10 as (an extremely good actively stabilized interferometer). The resulting precision, as defined in §2d, is shown in figure 7*a*. The step size was then fixed and the scan length was varied between 2 and 40 periods, shown in figure 7*b*, for the same jitter amplitudes. Both panels of figure 7 exhibit the same trend: if we want to increase the precision by a factor of two, we need to either decrease the step size by a factor of four or sample four times as many periods. This is not surprising, since we could have repeated the measurement four times instead, decreasing the standard deviation of the mean by a factor of $\sqrt{4}$. As mentioned earlier in §2d, what actually matters is the *number* of points with which we sample the sideband, not how we distribute them. Interestingly, even if we oversample, i.e. we sample with a step size smaller than the magnitude of the temporal jitter, we still increase the precision at the same pace. However, physical limitations in the piezo-controlled delay stage and feedback loop mean that we cannot decrease the step size indefinitely. When the physical step size limitation is hit, we can still sample more periods to further increase the precision. This also has the benefit of increasing the frequency resolution in the Fourier analysis. Since the highest frequency of interest is around 2*ω*, frequencies resolved above it are not usually of interest unless higher multi-photon processes are studied. In general, we found it is optimal in terms of absolute precision and acquisition time to sample at least 10 periods with a step size of 100 as or less, after which the absolute gain becomes very slow. It is important to note that the modelled temporal jitter provides an upper limit of the experimental jitter. For instance, vibrations on a time scale much shorter than the acquisition time will be averaged out in an experiment.

### (b) Influence of statistics

Here, the influence of the statistical noise and background fluctuations is evaluated. For each sampling point, we acquire a finite number of electrons that make up the sideband signal at each delay step. An error due to statistical noise (fluctuations in detecting electrons) is introduced from a Poisson distribution *P*(*σ*^2^_*s*_ = *S*(*τ*)).

**Figure 8.**
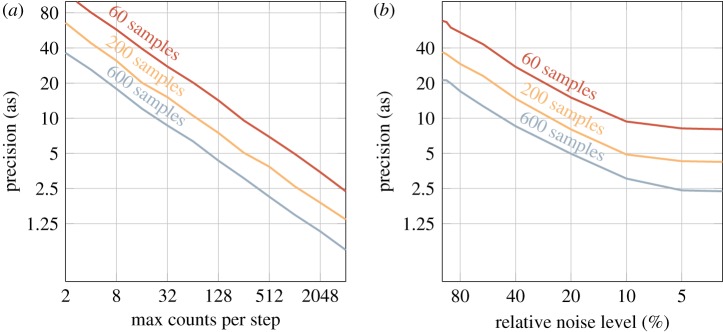
RABITT precision extracted from different sets of 1000 Monte Carlo simulations. (*a*) The maximum sideband amplitude was varied between 2 and 4086, and the Poisson noise was added to simulate the uncertainty of counting electrons in each delay step of the spectrogram. The simulations were repeated for a sample count of 60, 200 and 600 samples with a step size smaller than the Nyquist threshold for 2*ω*. (*b*) Precision as a function of relative background noise. The maximum sideband amplitude was set to 500 counts and the noise level was varied between 100% and 2% for the same three cases of sampling points.

In figure 8*a*, the precision is studied by varying the maximum sideband signal *S*(*τ*) from 2 to 4096 counts (which means that the sideband oscillates between 0 and the maximum signal). No background noise is taken into account yet. The trend is the same as in figure 7*a*,*b*, which again is expected since we would decrease the standard deviation by a factor of $1/\sqrt{N}$, where *N* is the number of measurements.

However, once a background noise is added, the precision is strongly influenced by the level of noise. In figure 8*b*, the sideband was set to oscillate between 0 and 500 counts, and the background noise was simulated as a white noise, drawn from a uniform distribution between 0 and 100% relative noise level. The precision improves at the same rate as the relative noise level decreases, up to the mark of 10% relative noise. This is where the precision reaches the background free limit; see the 500 counts mark in figure 8*a*. These simulations point out that the highest precision can be achieved either by acquiring data with signal-to-noise ratio of approximately 10 or by filtering the background noise by Fourier analysis.

### (c) Realistic RABBIT measurement

**Figure 9.**
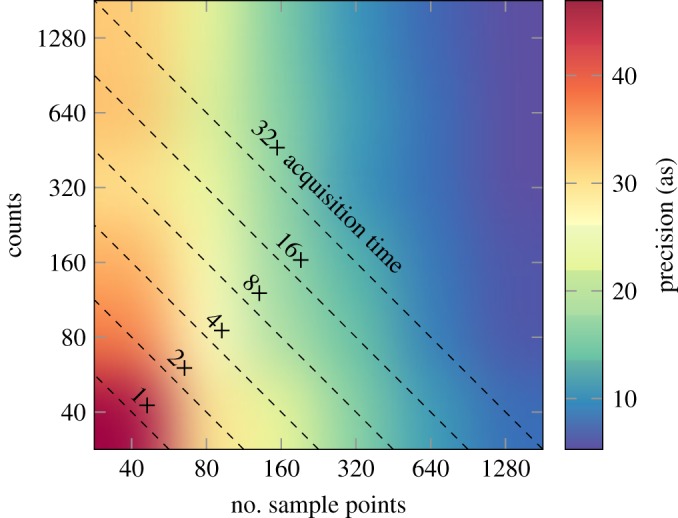
RABBIT precision as a function of number of sampling points and maximum allowed sideband oscillation measured in counts. Each diagonal line represents a line of constant acquisition time in a real experiment. The simulations were run with a jitter of 50 as, including statistical noise and a background level of 10%.

In a real experiment, we have an acquisition time to account for as well. A RABBIT spectrogram consists of many photoelectron spectra taken at different pump–probe delays, meaning the total acquisition time is the acquisition duration of each spectrum times the number of delay steps. Hence, we have to weigh the total number of sampling points against the time spent at each point, which affects the number of acquired counts in a single spectrum. To pinpoint the optimal conditions given a certain r.m.s. of the pump–probe delay jitter, we ran the same Monte Carlo simulations as above, mapping the two-dimensional parameter space of *acquired* counts and *number of sampling points* for a jitter r.m.s. of 50 as and 10% relative background noise. The result is shown in figure 9, where the precision is indicated by the colour map. As the scales are logarithmic, the diagonal lines correspond to a constant acquisition time, i.e. if we spend double the amount of time on acquiring counts on each step, we only sample half the number of oscillation cycles. Each point in the figure then corresponds to a RABBIT measurement. As shown, it is advantageous to spend the duration of the measurement on primarily sampling as many points of the sideband oscillation as possible, and only secondarily to acquire more counts at each sampling point. For a certain acquisition time, the absolute value of which naturally depends on the count rate of the experiment, the precision can vary as much as three times depending on whether counts or points are prioritized (see the line labelled ‘32 × acquisition time’). As indicated in figure 7, a 2 × reduction in jitter r.m.s. increases the precision by the same amount at no acquisition duration cost. The best way to increase precision, but certainly the most challenging, is to improve the stability of the interferometer.

## 5. Conclusion

In this work, we performed a comprehensive analysis of the RABBIT technique by investigating the influence of the properties of the light fields and discussing the statistical effects inherent to any measurement. We pointed out two predominant factors often limiting the temporal accuracy and precision to 5–10 as. The femtosecond chirp influences the phase retrieval and should be controlled and accounted for as much as possible. The long-term temporal stability of the controlled delay between the XUV and IR fields by the interferometer can be a severe limitation for the precision of a measurement without an active stabilization loop.

Monte Carlo simulations offer a solid tool to devise optimal strategies for data recording depending on the available acquisition time. The simulations show that the total number of sampling points of the sideband oscillation determines the precision of the phase retrieval, as long as the sampling rate is high enough to resolve the 2*ω* oscillation. Choosing a step size smaller than the magnitude of the temporal jitter, but higher than the physical limitation of the piezo-actuator, increases the fit accuracy. Fourier analysis can be used to filter the data, which can be of great importance if the background noise is above 20%. Below 20%, the background noise has a negligible effect. Optimal precision is found for the highest number of sampling points, not the highest number of counts.

To push the temporal accuracy/precision below 5 as, further studies and developments should be done. The simple interference equation (equation (2.2)) should be extended to include the femtosecond chirp, in order to account for the intrinsic variation in the periodicity. The fundamental laser intensity ought to be monitored in order to filter data. In addition, further studies of the influence of the pump and probe geometries in the interaction volume, including possible spatio-temporal couplings, are required. Finally, it is worth mentioning that our conclusions are in line with previous works done on streaking (e.g. [25]), and confirm the robustness of cross-correlation techniques, including the PROOF (phase retrieval by omega oscillation filtering) technique [21], to retrieve temporal information at the attosecond time scale.

## Data accessibility

This article has no additional data.
